# Guild Vertical Stratification and Drivers of Bat Foraging in a Semi-Arid Tropical Region, Kenya

**DOI:** 10.3390/biology12081116

**Published:** 2023-08-10

**Authors:** Ana Rainho, Diogo F. Ferreira, Beryl Makori, Michael Bartonjo, Miguel Repas-Gonçalves, Stanley Kirakou, Florah Maghuwa, Paul W. Webala, Ricardo Tomé

**Affiliations:** 1 cE3c—Centre for Ecology, Evolution and Environmental Changes & CHANGE—Global Change and Sustainability Institute, Departamento de Biologia Animal, Faculdade de Ciências, Universidade de Lisboa, 1749-016 Lisbon, Portugal; 2 CIBIO-InBIO, Research Centre in Biodiversity and Genetic Resources, BIOPOLIS Program in Genomics, Biodiversity and Land Planning, CIBIO, Campus de Vairão, University of Porto, 4485-661 Vairão, Portugal; 3 The Pangolin Project, P.O. Box 15156, Langata 00509, Kenya; 4 Mammalogy Section, National Museums of Kenya, P.O. Box 40658, Nairobi 00100, Kenya; 5 Strix, Rua Sousa Aroso, 556—4º D—trás, 4450-286 Matosinhos, Portugal; 6 Environment and Sustainable Development Department, Kenya Electricity Generating Company, P.O. Box 47936, Nairobi 00100, Kenya; 7 Department of Forestry and Wildlife Management, Maasai Mara University, P.O. Box 861, Narok 20500, Kenya; 8 The Biodiversity Consultancy, 3E King’s Parade, Cambridge CB2 1SJ, UK

**Keywords:** Africa, bat conservation, drylands, landscape management, species richness

## Abstract

**Simple Summary:**

Africa faces significant challenges in balancing economic and social development while preserving its natural resources. However, we still have much to learn about the diverse bat community on the continent, especially in drier ecosystems. In our study, which was conducted in a semi-arid region of Kenya, we aimed to provide detailed information on the factors that influence the number and activity of bats. We used acoustic sampling and other methods to assess bat activity at different heights above the ground. We surveyed 48 ground-level sites and two sites on meteorological masts 20 and 35 m above the ground. We identified over 20 bat species, including one species of concern for conservation. Our analysis showed that different variables affect bat activity. The low-flying bat species were influenced by habitat variables, whereas the high-flying species depended more on weather conditions. Our study highlights the richness of bat populations in semi-arid environments and emphasizes the need for conservation measures. Climate change, land management, and development projects threaten bat diversity and their habitats. It is crucial to implement effective management strategies to protect these species. Our findings contribute to the development of conservation efforts for bat populations in Africa and beyond.

**Abstract:**

Africa faces significant challenges in reconciling economic and social development while preserving its natural resources. Little is known about the diverse bat community on the continent, particularly in drier ecosystems. A better understanding of the bat community will help improve and inform the management of these ecosystems. Our study aimed to provide detailed information on the main drivers of bat richness and activity at three different heights above the ground in a semi-arid region of Kenya. We assessed how bat activity varied with space and height using acoustic sampling and complementary methods. We sampled 48 sites at ground level and two sites on meteorological masts at 20 m and 35 m above the ground. We recorded more than 20 bat species, including one species of concern for conservation. Our models showed that the use of space varies with bat guild, creating trade-offs in the variables that affect their activity. Low-flying bat species are mostly associated with habitat variables, whereas high-flying species are more dependent on weather conditions. Our study highlights the richness of bat assemblages in semi-arid environments and emphasizes the need for management measures to protect bat diversity in the face of habitat degradation caused by climate change, land management, and development projects.

## 1. Introduction

Due to the ever-increasing human population, Africa is confronted with a multitude of challenges in its quest to improve the living conditions of its people while at the same time preserving its unique natural resources and environment [1,2]. Much of Africa’s surface is now affected by human land use, resulting in significant biophysical disturbances to natural habitats, including large-scale land conversion, industrial activity, and infrastructure development [3,4,5]. Combined with the myriad negative impacts of climate change, these effects are leading to unprecedented biodiversity losses [6,7,8] and disruption of the healthy functioning of the ecosystem [9,10].

Infrastructure development (and related projects) is one of the key drivers of biodiversity loss [11,12]. Therefore, an early risk assessment and associated spatial planning, as well as the design of appropriate mitigation measures, are crucial to avoid or minimize potential impacts on biodiversity. However, countries from the Global South often face the challenges of rapid development, limited ecological data for effective planning, and weaker policy enforcement [1,13]. In such regions, gathering comprehensive ecological data on poorly studied animal groups is crucial to support the formulation of effective mitigation policies, assist governments and businesses in implementing policies, and achieve biodiversity goals [13,14].

Africa is particularly vulnerable to the impacts of climate change, with experts predicting an increase in the intensity of droughts linked to the El Niño-Southern Oscillation phenomenon [15,16]. As the most arid continent on earth, 61% of sub-Saharan Africa is comprised of drylands, and most African nations are currently facing a shift towards drier conditions [17]. Human populations in these regions often rely on rainfed agriculture and struggle to adapt to the harsh conditions, making them among the poorest in Africa [18,19], thereby leading to conflicts between people and the biodiversity of these regions [18,20]. Natural arid and semi-arid lands (ASALs) have unique flora and fauna of great conservation concern, and support high biological diversity at multiple levels [21]. However, these regions are often assumed to be habitat- and species-poor and suffer low environmental impact. These factors make ASALs common targets for development projects.

Bats (Mammalia: Chiroptera), the second most diverse group of mammals globally [22], are relatively understudied in Africa. They are critical components of mammalian fauna in the ASALs, comprising one of the most diverse and successful groups of mammals in ASALs [23]. Bats’ remarkable versatility, their sensitivity to environmental changes, and their critical role in seed dispersal, pollination, and pest control [24,25] make them an invaluable indicator group for studying the effects of habitat disturbance on arid tropical ecosystems [26,27,28]. Bats are highly vulnerable to habitat changes caused by human activities and face numerous threats that endanger their survival [29,30,31]. The potential decline and extinction of bats could have far-reaching ecological consequences, impacting not only biodiversity but also forestry and agriculture [32]. Despite their vital role in sustaining healthy and balanced ecosystems [33], conserving bats remains a considerable challenge. Further research is needed to understand the ecological roles of bats in ASALs, as well as their ability to adapt to environmental changes. This knowledge is essential for designing effective conservation measures and mitigating risks arising from human activities [23].

Some studies have investigated African bat responses to seasonal and habitat changes in dry regions [34,35], showing that bat activity declines during the dry season, and that land cover associations are guild- and season-specific. Other studies suggest that bats may also segregate their niche based on height, with traits such as diet, wing morphology, and echolocation playing key roles in determining their vertical stratification [36]. This phenomenon has been extensively studied in rainforests, where vegetation complexity and height increase the vertical niche availability [37,38,39]. In Africa, the vertical stratification of bats has been studied in a West African forest, focusing solely on frugivorous bats [40]. Taken together, these studies show that bat species composition changes across different forest strata, with bat activity being generally higher at the canopy level. Additionally, they show that forest fragmentation and the presence of water bodies appear to dilute this stratification. Nevertheless, there remains a dearth of information on how bat species use vertical spaces in open areas.

Understanding the seasonal patterns of bat activity and the vertical stratification of their activity is needed, e.g., to inform the early risk assessment of wind power projects and their spatial planning, including site selection and the design of appropriate mitigation measures that are crucial to avoid or minimize potential impacts on bats. In addition to informing wind-energy development, understanding these patterns may provide managers with insights into the timing of potential land management stewardship actions, minimising any negative impacts on bats [12,41]. Furthermore, understanding the ecological processes driving seasonal patterns of bat activity may provide insights into the adaptive response of bats to climate change [42].

In this study, we employed acoustic sampling and other complementary methods to investigate how environmental and landscape variables influence the occurrence, foraging behaviour, and vertical space use of five bat guilds in a semi-arid tropical region over the course of a year. We hypothesised that due to the distinct strategies that each guild uses to exploit their prey and the way they use the vertical dimension, there is a trade-off in the variables that drive their activity. Therefore, we predicted that the activity of low-flying species is driven mainly by habitat variables (e.g., land cover, terrain, and water availability) [34]. In contrast, we predicted that the activity of higher-flying species is mainly driven by weather and other environmental variables, such as wind speed and moon phase [43]. Taking the drastic seasonal weather changes typical of ASALs, we also predicted that humidity and wind speed influence the activity of high-flying bats throughout the year.

## 2. Materials and Methods

### 2.1. Study Area

**Figure 1:**
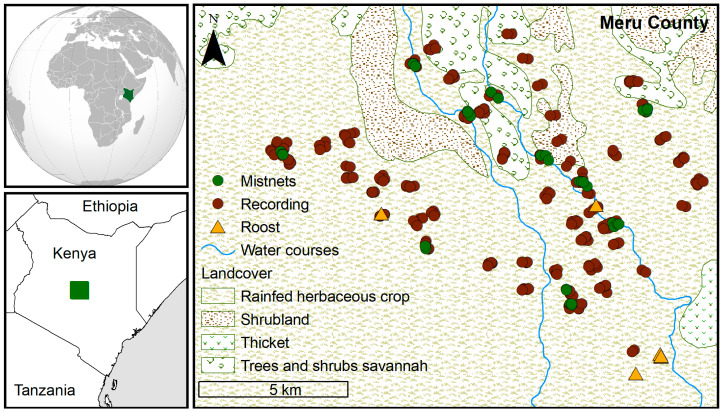
Map showing the spatial arrangement of sampling sites. Inset shows the study area location in Kenya and Africa.

**Figure 2:**
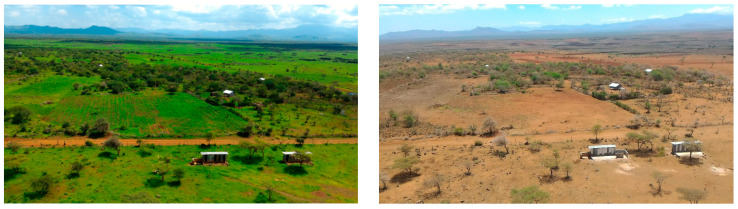
View of the study area in November 2016 (short rainy season, (**left**)) and September 2017 (end of the long dry season, (**right**)).

The study area, situated in Meru County (0.27–0.39°, 37.61–37.78°), Kenya (Figure 1), is a predominantly flat region with altitudes ranging from 1100 m to 1300 m. It has a semi-arid climate characterized by mild year-round temperatures. During our study, temperatures ranged from 19 to 27 °C, with the highest monthly average occurring in March. Humidity levels were highest in November and lowest in January, and they exhibited an overall decreasing trend until early October when they reached a new low. The wind speed peaked in August and reached its minimum in December and January. The area experienced two distinct rainy and dry seasons, resulting in significant seasonal changes in ground cover (Figure 2). The vegetation primarily consisted of *Vachellia-Commiphora* deciduous bushland and thicket, with some areas of rocky terrain. Tall trees were scarce, mostly found near streams, and there were no permanent water bodies. Subsistence farming, including crops like beans and maize, was practiced on cleared stretches of land, along with cattle grazing and some human settlements.

### 2.2. Bat Sampling

Bats were systematically surveyed using acoustic methods, both at ground level and height. Additionally, to ensure comprehensive coverage of the study area and adhere to best practices [44], we also utilized two complementary methods: (1) opportunistic consulting with local communities to gather information on bat occurrences and roosts, and (2) capturing bats using mist-nets.

We selected mist netting locations to maximize coverage of the study area, targeting patches of native woodlands or watercourses that are known to maximize bat capture rates [45]. At each sampling site, we deployed four mist nets (12 × 2.5 or 3.2 m, 16 mm mesh, Ecotone) at ground level. Nets were opened 30 min before sunset, left open for approximately 3.5 h each night, and checked at intervals of ca. 15 min. All the captured bats were identified, measured, photographed, and released at the point of capture. Identifications followed Patterson and Webala [46] and Happold and Happold [47], with the following remarks: (a) all the Pteropodid bats we captured had two post-dental ridges and forearm below 61 mm (Table S1), and were thus classified as *Epomophorus minimus* [48]; (b) as the classification of the East African *Rhinolophus landeri* is still uncertain, we refer to this clade as *Rhinolophus* cf. *landeri*; (c) we captured two species of *Mops*, with *M. pumilus* being significantly smaller than *M. condylurus*. However, their calls are indistinguishable, so we considered them a single phonic type; and (d) based on Musila et al. [49], we recognized the Dark-winged Lesser House Bat (*Scotoecus hirundo*) in our captures. However, the dark-winged forms of *Scotoecus* are difficult to differentiate and require molecular revision. Given the still pending taxonomic uncertainty for the group, we kept its classification as *Scotoecus* sp.

Reference calls from hand-released bats were recorded to create a reference call library to facilitate the acoustic identification of bats present in the study area. The bat nomenclature has been updated following recent publications [50,51,52].

We conducted ground acoustic sampling at 48 sites distributed throughout the study area during three separate field surveys: November–December 2016, May 2017, and September–October 2017. Seven to nine sites were sampled each night during the first three hours after dusk. Two-point counts were performed simultaneously at each site, each lasting for 5 min. All bat passes were recorded in wave format using an M500 USB Ultrasonic Microphone attached to a tablet (MS Windows Surface) operating the BatSound Lite software (Pettersson Elektronik AB, Uppsala, Sweden) at a sampling frequency of 384 kHz. The location of each point count pair was obtained using GPS, and the minimal distance between pair point counts was 400 m to avoid pseudo-replication. Land use, moon phase, and weather conditions (wind, cloud cover, and temperature) were recorded at each point count. Sampling was performed only in favourable weather conditions, avoiding heavy rain or foggy nights. All recorded calls were analysed using the BatSound v.4.21 Pro sound analysis software (Pettersson Elektronik, Uppsala, Sweden). The call characteristics, LowFreq (lowest apparent frequency), FreqMaxPwr (frequency of maximum amplitude), MaxFreq (highest apparent frequency), the interval between calls, and call shapes were extracted using the cursor lines. Identification was performed to the lowest taxonomic level possible by comparison between the measured values and the features of the calls of known species available in our database of African bat calls (from bat captures in mist nets and roosts) and with data available in the literature [47,53,54,55,56]. Several species were identified only to the genus level (e.g., *Mops* sp. and *Nycteris* sp.)

Bat activity was sampled high above ground using four automatic recording stations (Pettersson D500X; Pettersson Elektronik AB, Uppsala, Sweden; 500 kHz sampling rate, 16-bit resolution). Each pair was installed at two sites 20 m and 35 m above the ground, using existing meteorological masts. Sampling was performed between 29 November 2016 and 4 October 2017, automatically starting recordings at sunset, and stopping at sunrise. The recorders were synchronized to facilitate the identification of instances where the same bat call was detected at both heights. The input gain of the automatic recorders was set at 45 and the trigger level was set at 28 with no pre-triggering. The recordings lasted for 3 s and were spaced by a period of at least 5 s to avoid recording the same bat call in different files. We used Sonobat batch scrubber vs. 5.1 to exclude all files with no bat calls. Seventy-three sound metrics were automatically measured in eight call pulses recorded in each file using Sonobat 3.1p [57]. Calls were visually inspected, and metrics were averaged using a file whenever the values were consistent. Some of the measured sound metrics, namely call duration, LowFreq, FreqMaxPwr, Fc (characteristic frequency), FreqKnee (frequency at which the initial slope of the call most abruptly transitions to the slope of the body of the call), and Bndw20dB (the total bandwidth covered from the point of the call 20 dB below and before the point of maximum amplitude and the point of the call 32 dB below and after the point of maximum amplitude of the call), were further investigated using graphical and statistical analysis. Calls were identified to the lowest taxonomic level possible following the procedure described in the previous section.

### 2.3. Environmental and Landscape Descriptors

Several environmental and landscape descriptors were tested to assess their importance as drivers of bat activity in space and time (Table 1). Air temperature and absolute and relative humidity were recorded continuously using a HOBO Pro Series meteorological data logger (Onset Corporation) installed at a height of 20 m. In addition, wind data were recorded at the same site and height using an NGR wind assessment system. Landcover descriptors were derived from data retrieved in the field or Earth Observing System Data and Information Systems like the Landsat and the Shuttle Radar Topography missions (Table 1).

### 2.4. Data Analysis

We categorized all recorded bat species into six guilds based on published data on morpho-ecological traits, echolocation, and flight characteristics [35,46,47,54,58,59,60] (Table 2). All analyses were conducted in R v4.2.3 software [61], and all models were fitted with the “lme4” package [62]. Each model’s partial effects—estimated change in the response variable for each unit change in the independent variable, were obtained using the “Effects” package v. 4.2-2 [63,64].

To account for the differences in sampling effort, spatial distribution, and the nature of the data collected at different heights (occurrence and activity; see sections below), we evaluated differences in species richness between different heights through sample-size-based sampling curves following the procedure proposed by Chao et al. [65], using the package iNEXT [66]. Rarefaction was determined using sampling-unit-based incidence data (q = 0), as the mean of 500 replicate bootstrapping runs to estimate 95% confidence intervals. The sample size was extrapolated to 20 in both the habitat types sampled at ground level (~2 m): dense woodlands (N = 12) and farmlands (N = 14).

#### 2.4.1. Ground-Level Bat Occurrence

To investigate the relative effects of environmental and landscape variables on the occurrence of bats, while considering data from the different sampling methods (roost surveys, mist-netting, and ground acoustic) we used binomial generalised linear mixed models (GLMM) with logit as a link function. Separate models were used for each bat guild (Table S3). Guilds were only modelled when more than 30 presences were recorded, thus excluding the lowest flying guild Low_6 (Table 2). In all models, the occurrence of a given guild was used as the dependent variable, with the environmental and landscape metrics as independent variables (Table 2). Log-transformed sampling effort was included as an offset and the season was incorporated as a random term.

Correlation between variables was assessed using the Spearman correlation coefficient (Table S2), and one of the correlated variables (*r* > 0.7), usually the one with less biological relevance was excluded from the analyses [67]. After a graphical exploratory analysis, we tested each predictor and checked for non-linear relationships using univariate GLMM modelling. Variables with a *p*-value of over 0.3 were excluded from further analysis [68]. The most parsimonious models were selected based on their AICc values and the significance of the variables included in the model [69]. Whenever the variance of the random variables approached zero, the models were adjusted using generalised linear models (GLM) instead. Finally, we used variance inflation factors (VIF) to test for multicollinearity among variables in our final models [70]. However, no signs of multicollinearity were detected.

**Table 2:** Bat guilds considered in this study. The approximate flying height was estimated based on the authors’ expertise and available published information [35,47,58,59,60]. These values are provided as indicators and should be interpreted with caution. Guild species’ detection distance from Monadjem et al. [71]. Some species (in brackets) were not detected in our study area and are included solely for reference, due to the lack of specific information. N/A—No published information available.

	Guild	Taxa	Approx. Flying Height (m)	Mean Recording Distance (m)
High-flying bats	High_1	Monospecific guild, including only *Otomops harrisoni*	15–550	N/A
	High_2	All species of the family Molossidae, other than *O. harrisoni*	5–100	*M. pumilus:* 12.0 ± 2.00 *M. condylurus*: 15.0 ± 2.67
Medium- to high-flying bats	Medium_3	Genera *Scotophilus* and *Scotoecus*	3–15	[*S. dinganii*: 17.5] [*S.viridis*: 12.5]
	Medium_4	Genera *Pipistrellus*, *Afronycteris*, *Neoromicia, Nycticeinops* and *Miniopterus*	2–10	*A. nanus*: 5.8 ± 1.07 *N. schlieffeni*: 15.0 [*M. natalensis*: 5.0]
Low-flying bats	Low_5	Genera *Lavia*, *Cardioderma* and *Nycteris*	<3.5	N/A
	Low_6	Genera *Rhinolophus*, *Hipposideros* and *Triaenops*	<1.5	*H. caffer*: 0.2 ± 0.07 [*R. darlingi*: 2.0]

#### 2.4.2. High-Flying Bat Activity

We used negative binomial GLMMs with a log-link function to test the effects of several environmental variables on the night activity of each bat guild (see Table 2). Only guilds of high and medium-flying bats (1 to 4) were recorded at 20 m and 35 m heights. Guild 4 had very few observations and was thus not modelled. An index of bat activity (bat passes per hour [72]) was modelled with height and five environmental descriptors—temperature (°C), relative humidity (%), absolute humidity (g/m^3^), an illuminated fraction of the moon (%) and wind speed (m/s) (Table 1). As the two humidity measures were highly correlated, only the absolute humidity was used in the models. Wind proved to have a non-linear asymptotic relationship with bat activity and was thus log-transformed (log + 1). Season and site were used as random variables. The model-fitting procedure was described in the previous section.

## 3. Results

A total of 20 species belonging to nine families and six foraging guild types were recorded during the study (Table 3). One species, *Otomops harrisoni,* is globally classified as vulnerable [73]. We recorded at least three more species from the acoustic sampling, but these were unidentifiable at the species level. A total of 10 of the 20 species were recorded flying as high as 20 m above the ground. A particularly noteworthy observation was the recording of *Triaenops afer*, a low-flying species (guild Low_6), on one of the recorders deployed at 35 m.

**Figure 3:**
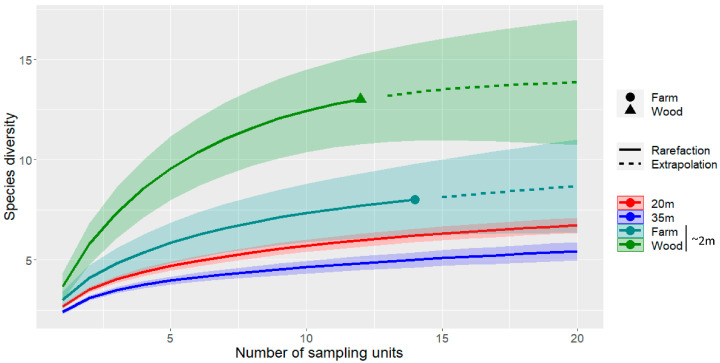
Sample-size-based (solid line) and extrapolation (dashed) sampling curves for species richness (q = 0) recorded using acoustic methods at three different heights (~2 m, 20 m, and 35 m), using two landcover types for ground sampling (~2 m): open farmlands (farm) and dense woodlands (wood). Shaded areas represent 95% confidence intervals.

Despite the reduced sample size of acoustic calls at ground level compared to those at higher elevations, it resulted in a much higher number of recorded species. When comparing species richness between the ground and height sampling, the results showed significant differences. Even when considering the different land-cover types sampled at ground level separately, these differences remained consistent. For example, at ground level, bat richness was nearly three times higher in densely wooded habitats than that recorded at 35 m elevation (see Figure 3). Although differences in richness were less noticeable in open habitats such as farmlands, bat richness remained significantly lower at 35 m height (Figure 3).

### 3.1. Ground-Level Bat Activity

**Figure 4:**
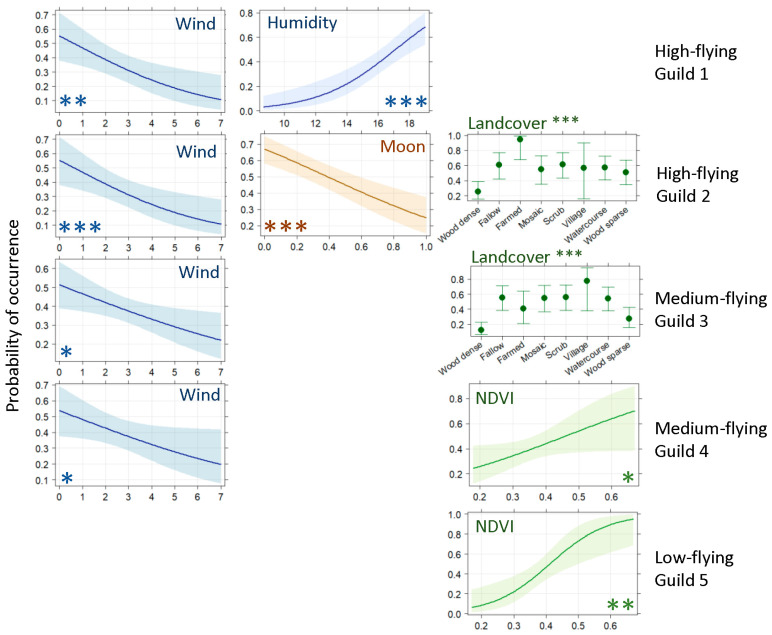
Binomial GLM models’ partial effects for the occurrence of each bat guild recorded at the ground level. Order of environmental variables from left to right and top to bottom: wind (Beaufort scale, from 0—calm to 7 high wind), relative humidity (%), fraction of visible moon (between 0—new and 1- full moon), landcover, and NDVI (index ranging from −1—no green biomass to +1—full green biomass; see Table 1 and Table 2 for further details on guilds and variables). Error bars and shaded areas represent 95% confidence intervals. Asterisks represent the significance of the effect (*** <0.001; ** <0.01; * <0.05). See Table S3 for the complete models.

The wind speed (Beaufort scale) was found to be a significant driver of foraging bat occurrence (Figure 4 and Table S3). While it did not have a significant effect on the low-flying Guild 5, all other guilds showed a similar trend of decreasing the occurrence of foraging bats at higher wind speeds. Landscape variables, such as NDVI and landcover, were found to drive bat occurrence in all guilds, except for the high-flying guild 1. The occurrence of the two lowest flying guilds modelled (Low_5 and Medium_4) was positively influenced by NDVI, indicating a preference for areas with greener vegetation. Both guilds Medium_3 and High_2 showed a preference for watercourses, with High_2 bats being more frequent in farmed areas and Medium_3 bats preferring urbanized areas and other open habitats.

Two additional variables were found to have an impact on the night occurrence of certain bat guilds in the studied region. An increase in the percentage of the visible moon was found to cause a decrease in the occurrence of molossid bats in guild High_2. Moreover, the occurrence of *O. harrisoni*, a unique member of guild High_1, was found to be positively influenced by relative humidity.

### 3.2. High-Flying Bat Activity

**Figure 5:**
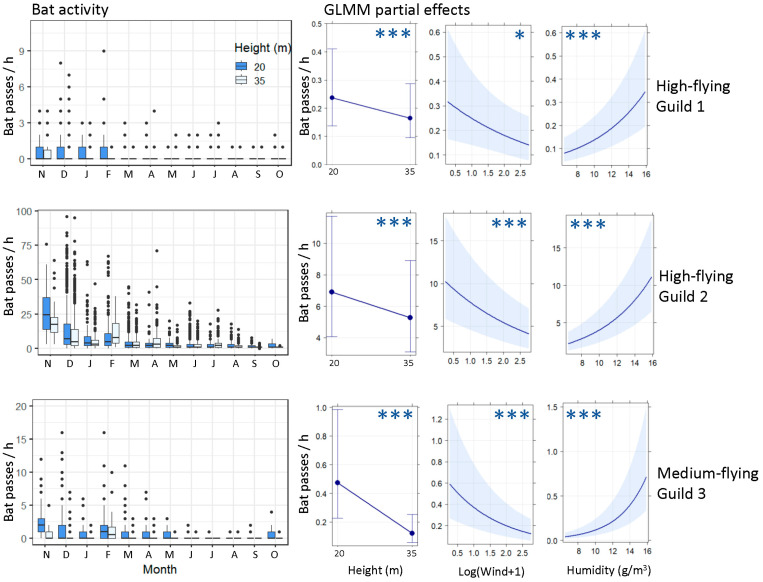
Bat activity at 20 and 35 m heights and its drivers throughout the year. The first column shows the monthly bat activity (bat passes/hour) of the three medium- and high-flying guilds at two heights from November 2016 to October 2017. The three columns on the right show each guild’s GLMM model partial effects of the main bat activity drivers: height (20 and 35 m), wind speed (m/s), and absolute humidity (g/m^3^), respectively. Error bars and shaded areas represent 95% confidence intervals. Asterisks represent the significance of the effect (*** <0.001; * <0.05). The activity of guild Medium_3 was also significantly and positively affected by temperature (not represented). All models include the site and season as random intercept values. See Table 1 and Table 2 for further details on guilds and variables and Table S4 for the complete models.

Wind, humidity, and height were significant descriptors of bat activity for the three guilds of medium- to high-flying bats, as depicted in Figure 5 and French Development Agency S4. Furthermore, temperature positively influenced the bat activity in guild Medium_3. Bat activity at 35 m was consistently lower than that at 20 m elevation, whereas activity increased under conditions of lower wind intensity and higher humidity.

The impact of wind speed on the occurrence and activity of guild High_2 was particularly apparent. An increase of 1 m/s in wind speed could lead to a 6% reduction in bat activity when all other variables were held constant. However, it is worth noting that even on nights with the highest recorded wind speed during this study (14.8 m/s), the overall bat foraging activity remained high (>6 bat-passes/hour) when the weather conditions were otherwise favourable (e.g., temperature > 24 °C and humidity > 14.5 g/m^3^ at 20 m elevation) and there was no moon.

## 4. Discussion

We used autonomous bat detectors and complementary methods to investigate how the occurrence, activity, and species richness of insectivorous foraging bats relate to landscape and environmental variables in a semi-arid landscape in Kenya. Despite employing different sampling strategies at ground and height, the results of both datasets revealed that bat guilds show a clear vertical stratification, with higher richness near the ground, particularly in wooded habitats. Low-flying species were recorded foraging almost exclusively at the ground level. High-flying species were recorded at the three sampled heights (~2, 20, and 35 m) but showed a decline in activity at 35 m above the ground.

Our predictions were supported by the findings that the activity of the second-lowest flyer guild (Low_5) was mainly driven by vegetation greenness (Figure 3), while the activity of the highest-flying guild (High_1) appeared to be constrained mainly by wind and humidity (Figure 3 and Figure 4). The trend was less clear in the remaining medium- to high-flying guilds, which seemed to be driven both by weather (mainly wind and humidity) and by landscape variables (Figure 3 and Figure 4). As observed elsewhere in Africa, the weather conditions observed between November and March—moderate air temperature, high humidity, and low wind speed [74,75]—provide the best conditions for the foraging activity of high fliers in this semi-arid area (Figure 5).

Free-tailed bats (Family Molossidae) fly high above the ground to exploit high-flying and migrating insects [76,77]. Therefore, it was unsurprising that this is the group most frequently recorded flying at 20 and 35 m above the ground in this study. Our study showed that molossids were the most active species at 35 m, even when general bat activity seemed to taper with height. Weather and other environmental conditions can influence insect abundance and activity, thereby influencing bat foraging activity [74,78]. No moon effect was found in most studied guilds. This finding supports the results of Musila et al. [79], who reported no evidence of lunar phobia in bats belonging to guild Medium_3 on the north coast of Kenya. The effect of the moon on the activity of the two most prevalent bat species in the region (*M. pumilus* and *M. condylurus*, guild High_2) was inconclusive in our study. While moon illumination had a minor but significant negative effect on the occurrence of this guild when sampled from the ground, no effect was found on its activity when measured at height. The effect of moonlight on insectivorous bat activity can be complex and varies depending on the species of bat, the ecological context, and the time of year [31,74,75,80]. To confirm if any lunar phobia occurs, further studies focusing on each *Mops* species are necessary.

Our study found that land cover was an important driver of bat occurrence in the medium-high flying guilds (High_2 and Medium_3). Land cover use varied across the two guilds, but both showed a lower occurrence in wooded habitats, particularly when the vegetation was dense (Figure 3). Instead, both guilds showed a preference for open habitats, including watercourses, which aligns with the well-established trend already documented in Africa [81,82]. Both guilds, which include species of the genus *Mops*, *Scotophilus,* and *Scotoecus*, were frequently detected in the villages of the area, even if there was some variability in the use of these human habitats. *Mops* and *Scotophilus* species commonly roost in buildings with the former often forming large colonies (pers.observ.; [54]). As a result, these species are considered to have the ability to exploit or at least adapt to urban areas [83].

The lower flying guilds (Medium_4 and Low_5) appeared to be less affected by the land cover types. Even when land cover was modelled as a single variable, both guilds showed a non-significant preference for densely wooded habitats, and in the case of Medium_4, also for farmed areas. The index of vegetation greenness was found to better reflect these species’ habitat preferences. The species in these guilds seem to use native habitat patches, such as wooded areas, watercourses, bushes, and thickets, that maintain green foliage even during the dry seasons. This finding is consistent with the findings of Hackett et al. [84], who found that green stands of *Acacia* trees were the most important natural arid habitat for insectivorous bats. Farmlands were found to be used by guild Medium_4, but only when the fields were at their greenest with crops (Figure 4). This may reflect the opportunistic behaviour of these vespertilionid and miniopterid species, which often target abundant crop pest species [25,33,85].

ASALs are highly vulnerable to habitat change and degradation, driven by multiple factors such as changes in agricultural and husbandry practices [86], and the construction of large-scale developmental energy projects, such as solar or wind farms. With an annual photovoltaic power potential of over 1800 kWp [87] and wind speeds reaching above 14 m/s, our study area, like many other high-altitude tropical arid and semi-arid regions, presents significant potential for the installation of solar and wind farms. However, while these environmentally friendly options are a clear improvement over fossil fuels, on-shore projects can still have both direct and indirect impacts on wildlife [88,89]. For instance, in addition to the habitat degradation during project installation, the operation of wind turbines can significantly decrease soil moisture, resulting in visible changes to vegetation, particularly in drylands [90]. The impacts of wind farms on bats have been extensively studied [12]. Our results suggest that wind projects can have a significant impact on the entire bat assemblage, affecting both medium- and low-flying species through habitat degradation and increasing the mortality rates of medium- and high-flying species due to collision and barotrauma. Furthermore, our models offer detailed information regarding the relationship between bat activity and weather variables, which can be valuable in informing risk-management strategies for such projects.

The conversion of extensive agricultural production to more intensive practices involving irrigation and the use of pesticides also poses a serious threat to insectivorous bat populations. In addition to the potential contamination of bats with agrochemicals [91], intensified agricultural practices can lead to a reduction in the availability of prey and roosts, particularly if they impact the native vegetation that remains [41,92]. Thus, potentially leading to a reduction in the activity and pest suppression services of bat species associated with these systems, such as *M. condylurus* and *M. pumilus* from guild High_2. Also, intensified livestock production can significantly impact arid and semi-arid ecosystems by exerting high pressure on native vegetation through browsing and trampling, resulting in habitat degradation and fragmentation [93]. The negative impacts of intensified agricultural and husbandry practices on biodiversity in arid and semi-arid regions highlight the crucial need to implement sustainable practices, such as those proposed by Maitima et al. [6] and Schurch et al. [94], which minimize harm to bats and other wildlife while also supporting human livelihoods.

Soil and land degradation due to human activities can be aggravated by climate change [95]. While low-flying bat species in our study area may have adapted to dry conditions [96], they still depend on patches of evergreen vegetation and tree cover to maintain prey abundance and support bat populations [97,98]. Several studies have already shown that the ongoing climate change has deleterious effects on the distribution of many species in some parts of the world [99,100] and has altered the migration patterns and reproductive timing of others [99,101,102]. ASALs in Africa may not be an exception. In the study area, bats from all guilds are present throughout the year. However, the decline in activity (Figure 5) suggests that most high-flying bats may shift to other areas during the dry-windiest months, most probably due to low prey availability [84]. Low-flying species may be unable to shift [96] and may be forced to endure harsh conditions. Other strategies, such as an increase in foraging periods and the use of larger foraging areas, have been described in low-flying species, like *Lavia frons* (guild Low_5), to cope with the lower prey availability associated with drier periods in ASALs [96]. The deterioration of landscape and climate conditions could cause a decline or even local extinction of bat populations in these arid areas. Either way, if bats become locally extinct or migrate and range extents are modified, this could have cascading effects on reproductive timing, predator-prey dynamics, and their ecosystem services on dry lands, further degrading these landscapes [33,85,103].

## 5. Conclusions

This study challenges the common idea that ASALs have very low species diversity and are, therefore, less vulnerable to impacts. Considering the African context [82] the study area showed a reasonably diverse bat assemblage. With the detection of unidentifiable echolocation calls, bat richness in the study area has the potential to exceed 20 species, including one species with a globally threatened status. Despite the sparse and simple vegetation structure, bats utilized the three-dimensional space in a complex manner, even if the activity is tapered with height. This vertical stratification proved to be consequential in the variables determining bat presence and activity. The species flying at lower altitudes showed a strong association with habitat variables, whereas those flying at higher altitudes were affected more by weather conditions.

Our study demonstrated the need for more discussions about management guidelines designed to minimize human impacts on bats, especially when considering land management and project development in ASALs. Maintaining or improving native bushlands and thickets should be a priority for ASAL managers and project developers in East Africa. As climate change escalates, changes in human activities, such as livestock husbandry, farming, timber harvesting, prescribed burning, or implementing development projects, such as solar and wind farms, will need to be carefully considered if the persistence of local bat species and other fauna is to be guaranteed.

## Figures and Tables

**Table 1:** Description of environmental and landscape descriptors used in the study.

Variables		Type, Units, and Classes	Source/Scale
Landscape Variables	Landcover	Land cover containing all habitat types categorised during fieldwork. Categorical: fallow, farmed, mosaic, pond, scrub, village, watercourse, wood dense, and wood sparse. Wood dense was used as a reference.	Recorded in the field
	NDVI	Normalised Difference Vegetation Index—a measure of green biomass for April. Continuous (−1 to 1). Available at: www.usgs.gov (accessed on 12 January 2018).	Derived from Landsat 8. 30 m
	DEM	Digital elevation model—a measure of altitude. Continuous, ranging from 1090 to 1288 m. Available at: http://www.cgiar-csi.org/data/srtm-90m-digital-elevation-database-v4-1 (accessed on 12 January 2018).	SRTM. 90 m DEM
	Distance to water	Distance to main watercourses in the study area (often maintained water in scattered puddles). Continuous, ranging from 0 to 4859 m.	Derived from AGID
Environmental Variables	Temperature	Ambient temperature sampled at the meteorological mast at Kandebene. Continuous, ranging from 19.3 to 27 °C.	Sensor at 20 m height
	RH	Relative air humidity was sampled at the meteorological mast at Kandebene. Continuous, ranging from 32.3 to 87.7%.	Sensor at 20 m height
	AH	Absolute air humidity was sampled at the meteorological mast at Kandebene. Continuous, ranging from 6.5 to 15.9 (g/m^3^)	Sensor at 20 m height
	WindClass	A measure of wind speed at ground level, using the Beauford scale (0—calm to 7—high wind speed).	Recorded in the field
	Wind speed	A measure of wind speed was sampled between November 2016 to October 2017. Continuous, ranging from 0.3 to 14.8 m/s.	Sensor at 20 m height.
	Moon	Fraction of the moon visible from November 2016 to October 2017. Continuous, ranging from 0 (new) to 1 (full moon). Available at: www.timeanddate.com/moon/kenya/isiolo (accessed on 12 January 2018).	Time and date website

**Table 3:** Bat species detected in the study area by survey type. Guild-type acronyms and descriptions are presented in Table 2. The echolocation calls of the *Mops* species could not be distinguished and are thus shown in brackets. Since *E. minimus* does not echolocate, it was not acoustically sampled; therefore, it is denoted with a hyphen.

		Sampling Method				
Family/Species	Guild	Roost Search	Mist Net	Ground Acoustic	Height Acoustic	
					20 m	35 m
Pteropodidae						
*Epomophorus minimus*		x	x	-	-	-
Rhinolophidae						
*Rhinolophus fumigatus*	Low_6			x		
*R.* cf. *landeri*	Low_6			x		
Hipposideridae						
*Hipposideros caffer*	Low_6			x		
Rhinonycteridae						
*Triaenops afer*	Low_6			x		x
Megadermatidae						
*Cardioderma cor*	Low_5		x	x		
*Lavia frons*	Low_5		x	x		
Nycteridae						
*Nycteris* sp.	Low_5	x		x		
Molossidae						
*Mops condylurus*	High_2	x	x	[x]	[x]	[x]
*Mops pumilus*	High_2	x	x	[x]	[x]	[x]
*Otomops harrisoni*	High_1			x	x	x
Miniopteridae						
*Miniopterus* sp.	Medium_4			x	x	x
Vespertilionidae						
*Afronycteris nana*	Medium_4			x		
*Laephotis kirinyaga*	Medium_4		x	x	x	
*Neoromicia somalica*	Medium_4		x		x	
*Nycticeinops schlieffeni*	Medium_4		x	x		
*Pipistrellus hesperidus*	Medium_4			x	x	x
*Scotoecus* sp.	Medium_3		x	x	x	x
*Scotophilus andrewreborii*	Medium_3	x	x	x	x	x
*Vansonia rueppellii*	Medium_4			x	x	

## Data Availability

The data that support the findings of this study are available from the corresponding authors upon reasonable request.

## Supplementary Materials

The following supporting information can be downloaded at: https://www.mdpi.com/article/10.3390/biology12081116/s1, Table S1: Summary of the data recorded from bats captured during the study; Table S2: Spearman correlation matrices displaying the correlation values between continuous independent variables; Table S3: Explanatory models of bat-guild occurrence at the study area using ground-sampling data; Table S4: Effect of environment variables and height on the activity of high-flying guilds as recorded at four automatic stations.
