# Supplementary material for: Guild Vertical Stratification and Drivers of Bat Foraging in a Semi-Arid Tropical Region, Kenya
Source: Biology (Basel). 2023 Aug 10;12(8):1116. doi: 10.3390/biology12081116 (PMC10452385; doi:10.3390/biology12081116)
Supplement: Supplementary file 1 [file biology-12-01116-s001.zip › biology-2449626-supplementary.pdf]

Guild vertical stratification and drivers of bat foraging activity in a semi-arid tropical region, Kenya.

Supplementary materials

Table S1. Summary of the data recorded from bats captured in mistnets (Nets) or roosts (Roost). The gender of each bat is defined as female (F) or male (M), its age as young (Y) or adult (A), and reproductive status as having small (S), medium (M), or large (B) testes (T) or nipples (N). The presence of false nipples (FN) and eventual pregnancies (Preg) are also listed. The length of the forearm, noseleaf (Nleaf), tragus, ear, and tail are indicated in millimeters, while the weight is in grams. We measured and averaged the average frequency of maximum power (FMaxE, kHz), minimum frequency (FMin, kHz), duration (Dur, ms), and interval (Int, ms) from five pulses of echolocation calls recorded when bats were released. The shape of the pulses was classified as modulated (FM), constant (CF), or quasi-constant (QCF) frequencies.

Species	Method	Date	YY	XX	Gender	Age / Cycle	Forearm	Weight	Nleaf	Tragus	Ear	Tail	Recording	FMaxE	FMin	Dur	Int	Shape
Cardioderma cor	Nets	30/11/2016	0.2773	37.722	F	A / SN	54.8	24.5	11.4	16.4	37.7							
Cardioderma cor	Nets	30/11/2016	0.2773	37.722	F	A / SN	54.3	29.5	11.7	16.9	35.4		_204117 A	45.16	40.08	1.1	53	FM
Lavia frons	Nets	30/11/2016	0.2773	37.722	M	A / ST	56.6	20.5	22.8	24.6	40		_205847 A					
Cardioderma cor	Nets	30/11/2016	0.2773	37.722	M	A / ST	52.4	27.5	12.6	13.7	31.8		_210759 A	43.98	36.06	1.48	142.6	FM
Cardioderma cor	Nets	30/11/2016	0.2773	37.722	M	A / ST	57.1	29.5	13.4	17.6	36.8		_211613 A	49.42	39.4	2.94	60.6	FM
Cardioderma cor	Nets	30/11/2016	0.2773	37.722	F	A / Preg	58.9	34.5	13.1	19.4	34.4		_212147 A	49.2	38.46	1.18	30.6	FM
Cardioderma cor	Nets	30/11/2016	0.2773	37.722	M	A / MT	53.6	30	13	15.7	34.7		_212614 A					
Cardioderma cor	Nets	30/11/2016	0.2773	37.722	F	A / FN / MN	56	35.5	12.2	14.9	37.2		_212942 A	46.48	38.88	1.54	23.42	FM
Cardioderma cor	Nets	30/11/2016	0.2773	37.722	M	A / MT	55.1	29	13.1	16.2	33.4		_213437 A					
Cardioderma cor	Nets	30/11/2016	0.2773	37.722	F	A / FN / MN / Preg	54.8	32	13.1	14.1	37.4		_213936 A	48.3	38.8	0.41	24.17	FM
Cardioderma cor	Nets	30/11/2016	0.2773	37.722	M	A / MT	55.3	26	12.8	16.4	37.2		_214505 A					
Cardioderma cor	Nets	30/11/2016	0.2773	37.722	M	A / ST	56.4	29.5	13.2	17.2	37.5		_214912 A	47.1	37.58	2.22	67.46	FM
Cardioderma cor	Nets	30/11/2016	0.2773	37.722	F	A / SN	57.1	29	12.4	15.8	36.2		_215221 A	49.38	40.18	2	71.04	FM
Cardioderma cor	Nets	30/11/2016	0.2773	37.722	F	A / SN	54.4	24	12.5	14.5	33.4		_215716 A	49.58	40.06	2.86	61.46	FM
Lavia frons	Nets	02/12/2016	0.3141	37.726	M	A / ST	57.25	23.5	20.7	25.4	38		_201727 A	34.22	28.5	0.93	28	FM
Lavia frons	Nets	02/12/2016	0.3141	37.726	F	A / SN	59.6	26	21.6	24.7	42.6		_203312 A					

Species	Method	Date	YY	XX	Gender	Age / Cycle	Forearm	Weight	Nleaf	Tragus	Ear	Tail	Recording	FMaxE	FMin	Dur	Int	Shape
Cardioderma cor	Nets	04/12/2016	0.3500	37.676	F	A / SN	57.5	27.5	13.6	13.6	41.1		_193201 A	49.56	43.62	1.67	26.94	FM
Cardioderma cor	Nets	04/12/2016	0.3500	37.676	F	A / SN	54.6	27.5	15.1	15.1	33.9							
Cardioderma cor	Nets	04/12/2016	0.3493	37.676	F	A / SN	57	31.5	15.2	15.2	28.6							
Nycticeius schlieffeni	Nets	04/12/2016	0.3500	37.675	M	A / MT	32.9	8			8.3	40.2	_201044 A					
Lavia frons	Nets	04/12/2016	0.3493	37.676	M	A / ST	58.6	33.2	20.5	22.1	38.1		_201623 A	37.14	28.66	1.71	40.75	FM
Lavia frons	Nets	04/12/2016	0.3493	37.676	F	A / FN / SN	62	30.05	23.9	27.7	42.5							
Cardioderma cor	Nets	04/12/2016	0.3493	37.676	M	A / ST	52.6	28.5	10.8	12.4	29.8		_203704 A	47.28	42.82	0.6	25.97	FM
Lavia frons	Nets	04/12/2016	0.3500	37.675	F	A / SN	62.6	29	23.9	28.1	43.3		_215751 A	31.5	27.76	0.8	56.92	FM
Lavia frons	Nets	05/12/2016	0.3356	37.746	F	A / FN / BN	61	31.5	23.3	29.9	45.4		_195431 A	37.28	27.24	2.92	121.9	FM
Cardioderma cor	Nets	05/12/2016	0.3363	37.746	F	A / BN	57.4	31.5	13.6	33.8	13.7		_211748 A	43.86	38.08	1.44	235.7	FM
Lavia frons	Nets	07/12/2016	0.2941	37.679	F	A / SN	62.5	25.5	22.8	24.2	40.7							
Lavia frons	Nets	07/12/2016	0.2949	37.679	F	A / FN / BBN	61.3	25	25	26	40							
Lavia frons	Nets	07/12/2016	0.2941	37.679	M	A / ST	59.4	21	20.7	23.3	40							
Scotoecus sp.	Nets	07/12/2016	0.2949	37.679	F	Y	29.2	5				24.1						
Scotophilus andrewreborii	Roost	08/12/2016	0.2617	37.75	F	Y	41	28.5										
Lavia frons	Nets	08/12/2016	0.3226	37.636	F	A / FN / BN	62.8		24.9	27.8	45.7							
Lavia frons	Nets	08/12/2016	0.3226	37.636														
Lavia frons	Nets	08/12/2016	0.3226	37.636														
Lavia frons	Nets	08/12/2016	0.3227	37.636	F	A / FN / BN	62.5	30	23.2	26.5	43.8							
Scotophilus andrewreborii	Nets	08/12/2016	0.3235	37.636	M	A / ST	51.8	23.5				52.5						
Lavia frons	Nets	09/12/2016	0.3350	37.693	M	A / ST	56.1	18.5	21.9	25.5	39.5							
Lavia frons	Nets	09/12/2016	0.3357	37.692	M	A / MT	56.9	20	21.4	25.4	41.9		_195250 A	35.18	31.38	0.76	22.55	FM
Lavia frons	Nets	09/12/2016	0.3357	37.692	F	A / FN / BBN	60.8	28	22.1	25.3	40.3		_195713 A	35.06	28.76	1.58	52.67	FM
Cardioderma cor	Nets	09/12/2016	0.3355	37.692	M	A / ST	55.6	29	14	17.6	41.5		_203426 A	41.98	37.94	0.71	21.56	FM
Cardioderma cor	Nets	09/12/2016	0.3355	37.692	M	A / BT	56.6	27.5	15.3	19.7	40.1		_204007 A	42.7	37.82	0.96	25.25	FM
Cardioderma cor	Nets	09/12/2016	0.3342	37.693	M	A / MT	54.8	27	14.6	18.4	38.4							

Species	Method	Date	YY	XX	Gender	Age / Cycle	Forearm	Weight	Nleaf	Tragus	Ear	Tail	Recording	FMaxE	FMin	Dur	Int	Shape
Lavia frons	Nets	09/12/2016	0.3342	37.693	M	A / MT	58.7	21	21.4	28.4	40.1		_213645 A	33.24	31.02	0.62	33.41	FM
Epomophorus minimus	Roost	08/12/2016	0.2624	37.750														
Epomophorus minimus	Roost	08/12/2016	0.2614	37.750														
Scotophilus andrewreborii	Roost	09/12/2016	0.2564	37.743														
Lavia frons	Nets	12/05/2017	0.3360	37.745	M	ST	58.1	22	22.2	27.3	42.7							
Cardioderma cor	Nets	12/05/2017	0.3365	37.746	F	SN	56.9	26	12.7	17.9	37.9							
Epomophorus minimus	Nets	15/05/2017	0.3145	37.726	M	ST	57.6	33			16.8							
Epomophorus minimus	Nets	15/05/2017	0.3144	37.727	F	SN	57.9	41.5			17.9							
Epomophorus minimus	Nets	15/05/2017	0.3144	37.727	M	ST	59.7	40			17.1							
Epomophorus minimus	Nets	15/05/2017	0.3130	37.728	M	ST	60	45			18.6							
Epomophorus minimus	Nets	15/05/2017	0.3130	37.728	F	A / LN	57	36			16.9							
Cardioderma cor	Nets	16/05/2017	0.2770	37.723	F	SN	58	30	12.9	15.7	36.7							
Lavia frons	Nets	16/05/2017	0.2774	37.723	M	ST	56.8	21.5	21.4	27.7	41.2							
Lavia frons	Nets	16/05/2017	0.2770	37.723	F	A / FN / SN	61.8	30	21.6	29	41.6							
Cardioderma cor	Nets	17/05/2017	0.3501	37.676	M	A / ST	56.9	28	13.3	16.5	37.5							
Cardioderma cor	Nets	17/05/2017	0.3501	37.676	M	A / ST	57.3	29	12.3	16.9	35.8							
Lavia frons	Nets	17/05/2017	0.3501	37.676														
Lavia frons	Nets	18/05/2017	0.3226	37.636	F	A / BN / FN	62	29.9	22.9	28.5	41.4							
Lavia frons	Nets	20/05/2017	0.2949	37.679	M	ST	58.1	20.5	20.5	22.9	39							
Epomophorus minimus	Nets	21/05/2017	0.3015	37.738	F	SA / SN	56.3	34.5			16.7							
Scotophilus andrewreborii	Nets	21/05/2017	0.3015	37.738	M	A / BT	51.6	21		6.8	13.3	47.4						
Epomophorus minimus	Nets	21/05/2017	0.3015	37.738	F	A / BN	57.7	37			16.6							
Epomophorus minimus	Nets	21/05/2017	0.3016	37.737	M	A / MT	57.9	36			16.8							
Cardioderma cor	Nets	21/05/2017	0.3007	37.736	M	A / ST	54.3	28.5	13.2	11.2	30.3							
Epomophorus minimus	Nets	21/05/2017	0.3007	37.736	F	A / BN	57.2	33.5			16.7							
Epomophorus minimus	Nets	21/05/2017	0.3007	37.736														

Species	Method	Date	YY	XX	Gender	Age / Cycle	Forearm	Weight	Nleaf	Tragus	Ear	Tail	Recording	FMaxE	FMin	Dur	Int	Shape
Epomophorus minimus	Nets	21/05/2017	0.3019	37.736	M	A / BT	58.8	36.5			14							
Epomophorus minimus	Nets	21/05/2017	0.3019	37.736	M	A / BT	58	40.5			16.3							
Epomophorus minimus	Nets	21/05/2017	0.3019	37.736	M	J / ST	56.4	31			15							
Epomophorus minimus	Nets	21/05/2017	0.3019	37.736	F	A / SN	59.1	39			17.1							
Scotophilus andrewreborii	Nets	21/05/2017	0.3019	37.736	F	A /SN / Preg?	24.4						_213621 D	33.66	30.8	3.42	88.4	FM(i)
Epomophorus minimus	Nets	22/05/2017	0.3224	37.715	M	ST	56.7	41.5			16.6							
Cardioderma cor	Nets	22/05/2017	0.3224	37.715														
Scotophilus andrewreborii	Roost	12/05/2017	0.3048	37.666														
Molossidae	Roost	12/05/2017	0.3048	37.666														
Neoromicia somalica	Nets	26/09/2017	0.3500	37.676	F	A / SN	32.5	10			9.7	32.6	_193456 A	44.2	35	2.1	27.2	FM/CF
Lavia frons	Nets	26/09/2017	0.3493	37.676	M	A / ST	57.3	18.5	28.4	34.6	45.3		_195251 A	37.32	26.91	2.5	112.9	FM
Cardioderma cor	Nets	26/09/2017	0.3500	37.676	M	A / ST?	56.8	28.5	15.5	18.1	35.8		_211604 A	43.1	39.1	1.03	52.4	FM
Cardioderma cor	Nets	27/09/2017	0.3354	37.692	M	A / ST	53.4	26	13	16.8	37.2		_203142 A	44.2	38.3	1.9	49.2	FM
Lavia frons	Nets	28/09/2017	0.3362	37.746	F	A / FN / BN	60.5	22.5	21.4	26.9	40.9		_190603 A					
Lavia frons	Nets	29/09/2017	0.3396	37.701	M	A / ST	60.4	21.5	21.9	25.1	41.6		_193554 D	37	29.2	2	138	FM
Cardioderma cor	Nets	29/09/2017	0.3414	37.699	M	A / MT	56.5	28.5	14.8	14.2	33.6		_200246 D	51.3	42	2.3	62.3	FM
Lavia frons	Nets	29/09/2017	0.3415	37.699	F	A / FN / BN	60.9	23.5	22.3	25.4	41.7		_200401 D	33.6	28	1.05	117	FM
Cardioderma cor	Nets	29/09/2017	0.3415	37.699	M	Y / ST	56.7	28	13.7	15.5	34.6							
Epomophorus minimus	Nets	30/09/2017	0.3223	37.716	M	Y / ST	58	41			19.4							
Epomophorus minimus	Nets	30/09/2017	0.3223	37.716	M	A / ST	57.1	38			15.7							
Epomophorus minimus	Nets	30/09/2017	0.3223	37.716	F	F	60.8	41.5			19.2							
Epomophorus minimus	Nets	30/09/2017	0.3222	37.714	M	A / ST	58.3	39.5			19.7							
Epomophorus minimus	Nets	30/09/2017	0.3209	37.716	M	A / ST	58.9	41			17.8							
Epomophorus minimus	Nets	30/09/2017	0.3209	37.716	M	A / ST	56.6	43			15.5							
Epomophorus minimus	Nets	01/10/2017	0.3143	37.727	M	A / BT	56.3	39.5			15.9							
Epomophorus minimus	Nets	01/10/2017	0.3143	37.727	M	A / BT	59.5	42			17.5							

Species	Method	Date	YY	XX	Gender	Age / Cycle	Forearm	Weight	Nleaf	Tragus	Ear	Tail	Recording	FMaxE	FMin	Dur	Int	Shape
Epomophorus minimus	Nets	01/10/2017	0.3143	37.727	M	A / BT	55.7				12.8							
Epomophorus minimus	Nets	01/10/2017	0.3143	37.727	M	A / BT	56.9	35.5			15.4							
Epomophorus minimus	Nets	01/10/2017	0.3143	37.727	M	A / BT	57.6	40			17.2							
Epomophorus minimus	Nets	01/10/2017	0.3143	37.727	M	A / BT	56.2	37			15.2							
Epomophorus minimus	Nets	01/10/2017	0.3143	37.727	M	A / MT	57.6	31.5			16.9							
Epomophorus minimus	Nets	01/10/2017	0.3143	37.727	M	A / MT	57.9	38.5			16.9							
Nycticeius schlieffeni	Nets	01/10/2017	0.3143	37.727	M	A / BT	33	5.5			8	33.5	_200203 D	41.3	36	3.7	86.3	FM/CF
Nycticeius schlieffeni	Nets	01/10/2017	0.3143	37.727	M	A / ST	34	7			9.3	37.3	_203537 D	40.1	35.3	3.09	84.1	FM/CF
Hipposideros caffer	Nets	01/10/2017	0.3143	37.727	F	Y / SN	46.9				11.4	27.4	_200803 D	154.1	118	6.9	16.3	CF/FM
Laephotis kirinyaga	Nets	01/10/2017	0.3143	37.727	M	A / MT	28.2	4			7.1	23	_204639 D	40.7	38	3.4	88.4	FM/CF
Mops pumilla	Nets	01/10/2017	0.3144	37.726	M	Y / ST	20	9			5.4	16.8	_212651 D	22.5	19.2	6.4	129.5	QCF
Mops pumilla	Nets	01/10/2017	0.3144	37.726	M	A / ST	37	10			10.2	26.8	_214207 A	24.8	20.2	6.3	171.5	QCF
Mops pumilla	Nets	01/10/2017	0.3144	37.726	M	A / MT	40	12			13.1	30.1	_214756 A	23.1	19.1	5.4	56.7	QCF
Mops pumilla	Nets	01/10/2017	0.3144	37.726	F	Y / SN	35.3				9.1	24.9	_223702 A	21.9	19.3	10.2	444.0	QCF
Mops pumilla	Nets	01/10/2017	0.3144	37.726	M	A / BT	37.9	10.5			11	30.1						
Scotoecus sp.	Nets	01/10/2017	0.3144	37.726	F	A / BN / Preg	34.6	13			8.9		_224500 A	37.8	34	4.8	72.9	FM/CF
Mops condylurus	Nets	01/10/2017	0.3143	37.727	M	A / MT	44.6	16.5			12.5	37.2	_224705 A	24.5	19.3	5.7	92.3	QCF
Cardioderma cor	Nets	02/10/2017	0.2819	37.722	F	A / BN	56.7	29	13.9	20	33.9							
Cardioderma cor	Nets	02/10/2017	0.2819	37.722	F	A / BN	58.5	28	12.6	19.2	39.8							
Lavia frons	Nets	02/10/2017	0.2819	37.722	M	A / ST	59.2	20	20.8	28.1	44.9							
Cardioderma cor	Nets	02/10/2017	0.2815	37.722	M	A / ST	52		12.6	15.9	35							
Cardioderma cor	Nets	02/10/2017	0.2815	37.722	F	A / SN	56.1	28	14.7	18.6	36.3							
Cardioderma cor	Nets	02/10/2017	0.2815	37.722	F	A / BN	59.2	29.5	16	20.8	35.1							
Cardioderma cor	Nets	02/10/2017	0.2815	37.722	F	A / FN / BN	54.6	27.5	14.8	16.3	30.2							
Cardioderma cor	Nets	02/10/2017	0.2815	37.722	F	A / BN	56.4	28	16.3	21.1	33.4							
Cardioderma cor	Nets	02/10/2017	0.2815	37.722	F	A / FN / BN	58.9	28.5	14.5	22.2	32.3							

Species	Method	Date	YY	XX	Gender	Age / Cycle	Forearm	Weight	Nleaf	Tragus	Ear	Tail	Recording	FMaxE	FMin	Dur	Int	Shape
Cardioderma cor	Nets	02/10/2017	0.2815	37.722	F	A / BN	57	27	14.7	20.6	35.2							
Cardioderma cor	Nets	02/10/2017	0.2815	37.722	F	A / FN / BN	57.6		15.1	22.1	37							
Cardioderma cor	Nets	02/10/2017	0.2815	37.722	F	A / FN / BN	56.8	28.5	14.2	17.1	32							

Table S2. Spearman correlation matrices displaying the correlation values between continuous independent variables. The top table summarizes the variables considered when modeling bat guild occurrence at ground level, while the bottom table pertains to the environmental variables used when modeling bat activity at height. For detailed information on each variable, please refer to Table 2 in the main document. It is worth noting that the correlation values, except for one, are all below 0.7, indicating the absence of strong correlations among variables. The only exception is the correlation between Relative and Absolute humidity (highlighted in bold) among the variables measured at height. As a result, these two variables were never included together in the same model.

Ground variables	*RH*	*T*	*DEM*	*Dist_water*	*NDVI_Apr*	*Moon*
Relative Humidity (RH)	1					
Temperature (T)	-0.4235	1				
DEM	-0.11092	0.023305	1			
Dist to watercourses	0.00991	-0.01342	0.044561	1		
NDVI_April	-0.07732	0.090435	-0.21555	-0.304713	1	
Illuminated moon	-0.30516	0.214421	-0.21384	0.00019854	0.1473429	1

Height variables	*T*	*RH*	*AH*	*Moon*	*Wind*
Temperature (T)	1				
Relative Humidity (RH)	-0.33007	1			
Absolute Humidity (AH)	-0.04556	**0.89391**	1		
Illuminated moon	0.04281	-0.31806	-0.39416	1	
Wind speed	-0.40951	-0.03054	-0.13291	0.15832804	1

Table S3. Binomial generalized linear models of bat-guild occurrence at the study area using ground-sampling data. Guild 6 was excluded due to the low number of observations. Variable names and descriptions are indicated in Table 1, guild descriptions and acronyms in Table 2. Significant results (p-value < 0.05) are highlighted in bold.

	High-flying Guild 1			***High-flying Guild 2***			***Medium-flying Guild 3***			***Medium-flying Guild 4***			***Low-flying Guild 5***		
Fixed	ß	SE	p	ß	SE	p	ß	SE	p	ß	SE	p	ß	SE	p
Intercept	-8.031	1.583	<0.001	-0.727	0.357	0.041	-3.285	0.390	<0.001	-1.286	0.841	0.126	-6.926	1.420	<0.001
Wind Class	-0.336	0.122	**0.006**	-0.578	0.088	**<0.001**	-0.188	0.079	**0.017**	-0.221	0.112	**0.048**			
Moon				-1.815	0.394	**<0.001**									
RH	0.407	0.0.93	**<0.001**												
Landcover - Fallow				1.533	0.523	**0.003**	2.173	0.533	**<0.001**						
Farmed				3.931	1.118	**<0.001**	1.584	0.612	**0.010**						
Mosaic				1.278	0.526	**0.015**	2.153	0.543	**<0.001**						
Scrub				1.549	0.505	**0.002**	2.205	0.537	**<0.001**						
Village				1.359	1.035	0.189	3.194	0.974	**0.001**						
Waterline				1.379	0.474	**0.003**	2.122	0.511	**<0.001**						
Wood sparse				1.117	0.469	**0.017**	0.975	0.507	0.055						
NDVI										4.061	2.090	0.050	11.306	3.675	0.002

Table S4. Negative binomial generalized linear mixed models resuming the effect of environment variables and height on the activity of high-flying guilds as recorded at four automatic stations. The activity at 20 m was taken as a reference for the categorical variable height. Significant results (p-value < 0.05) are highlighted in bold.

	High-flying Guild 1			***High-flying Guild 2***			***Medium-flying Guild 3***		
Fixed	ß	SE	p	ß	SE	p	ß	SE	p
Intercept	-2.806	0.489	<0.001	-1.133	0.671	0.091	-3.466	0.510	<0.001
Height - 35m	-0.361	0.096	**<0.001**	-0.272	0.037	**<0.001**	-1.367	0.085	**<0.001**
Ab. Humidity	0.156	0.026	**<0.001**	0.171	0.010	**<0.001**	0.309	0.024	**<0.001**
Temperature				0.068	0.023	**0.003**			
Wind Speed	-0.325	0.132	**0.013**	-0.364	0.064	**<0.001**	-0.630	0.104	**<0.001**
Random	Var	SE	Groups	Var	SE	Groups	Var	SE	Groups
Season	0.286	0.535	4	0.205	0.453	4	0.465	0.682	4
Site	0.003	0.054	2	0.041	0.202	2	0.038	0.194	2
